# Role of Apyrase in Mobilization of Phosphate from Extracellular Nucleotides and in Regulating Phosphate Uptake in Arabidopsis

**DOI:** 10.3390/ijms262411857

**Published:** 2025-12-09

**Authors:** Robert D. Slocum, Huan Wang, Xingbo Cai, Alexandra A. Tomasevich, Kameron L. Kubecka, Greg Clark, Stanley J. Roux

**Affiliations:** 1 Department of Biological Sciences, Goucher College, Towson, MD 21204, USA; 2 Department of Molecular Biosciences, The University of Texas at Austin, Austin, TX 78712, USAgbclark@utexas.edu (G.C.); sroux@austin.utexas.edu (S.J.R.)

**Keywords:** apyrase, eATP, auxin, extracellular ATP, nucleotide salvaging, PSR, phosphate starvation-responsive, RSA, root system architecture

## Abstract

Apyrase (nucleotide triphosphate diphosphohydrolase, NTPDase; EC 3.6.1.5) functions in a variety of plant growth and developmental processes, as well as responses to pathogens, in part, by regulating extracellular ATP (eATP) concentrations. In this study, we investigated potential roles of apyrase in the recruitment of phosphate (Pi) from extracellular nucleotides in *Arabidopsis thaliana* seedlings that constitutively overexpress *apyrase 1* (*APY1*). Under Pi limitation, both WT and *APY1* seedlings had decreased Pi contents and a characteristic remodeling of root system architecture (RSA). This phosphate starvation response (PSR) was prevented by the uptake of Pi released through the metabolism of extracellular NTP, which occurred at a higher rate in *APY1* seedlings. *APY1* seedlings had higher Pi contents than WT seedlings on Pi-sufficient media supplemented with NTP and exhibited markedly increased LR and root hair (RH) formation. Genome-wide expression profiling revealed that this expanded RSA of *APY1* seedlings was correlated with the induction of >100 genes involved in regulation of auxin homeostasis, signaling, and transport, which previous studies have shown to be increased when *APY1* is overexpressed. APY1 regulation of [eNTP] and purinergic signaling may thus contribute to modulation of auxin responses, resulting in enhanced uptake of Pi from the medium, including Pi released via eNTP metabolism.

## 1. Introduction

Phosphorous is an essential nutrient for plant growth and development and is acquired from the soil. Concentrations of inorganic phosphate (Pi) in the soil are typically 1–2 µM [1] but high-affinity Pi transporters (PHT) in the root efficiently scavenge this nutrient and concentrate Pi in plant tissues to 10 mM or higher concentrations [2,3]. The vast majority of phosphate in soils exists in organic forms such as nucleic acids, free nucleotides, sugar-phosphates, phospholipids, and polyphosphates [4,5]. In many natural soils, recruitment of Pi from these sources into plants occurs via extracellular phosphatase (ecto-phosphatase) activities of associated ectomycorrhizae [5]. Under phosphate limitation, non-mycorrhizal plants like Arabidopsis also secrete a variety of extracellular phosphatases, such as purple acid phosphatases, to hydrolyze Pi from organic molecules for direct uptake [6].

In the rhizosphere, nucleic acids and nucleotides of various types represent a potentially significant source of P nutrition, under P limitation. Phosphate mobilization from extracellular nucleic acids through the combined activities of nucleases, such as the P starvation-responsive (PSR) RNase RNS1 [7], and ecto-phosphatase activities has been reported [8,9]. Free extracellular nucleotides (eNTP) may also serve as another potential source of Pi and can be released by damaged or decaying cells in the soil [10], by exudation from pathogen-infected roots [11], or via vesicle-mediated secretion or efflux from root cells [12,13]. In organic-rich soils, adenylate pools of up to 50 nmol/g soil have been reported and are tightly correlated with microbial biomass [14]. Estimates of free nucleotide concentrations are generally unavailable, but a free ATP concentration of ~45 nM was estimated for a defined soil one week after the inoculation of sterile medium with 10,000 cfu of a field flora [15]. Total nucleotides approaching micromolar concentrations may therefore be expected to occur in native soils. A major unanswered question is whether apyrase enzymes (NTPDases) can play a role in the recruitment of phosphate (Pi) from extracellular nucleotides in *Arabidopsis thaliana.*

The extent to which the salvaging of eNTP can help meet metabolic demands for cellular Pi is poorly understood. Although export of cellular ATP via a plasma membrane transporter has been reported [16], plasma membrane transporters responsible for nucleotide uptake have not been identified in plants [17]. Thus, mobilization of Pi from eNTP through intracellular metabolism is not possible. A role for secreted purple acid phosphatases in recruitment of Pi from dNTP [18] or ATP in soybean, bean, and poplar [19,20,21] has been demonstrated. Ecto-apyrase activities also function in nutrient salvaging from extracellular ATP (eATP). In potato, nucleobase uptake via the apoplastic salvaging of ATP is mediated by an ecto-apyrase, in coordination with other ecto-phosphatases and a nucleoside hydrolase [22]. The apoplastic salvaging of NTPs as a sole source of nitrogen was also shown to partially alleviate symptoms of N-limitation in Arabidopsis seedlings [23]. Additional studies support a role for ecto-apyrase activity in Pi mobilization from ATP. Thomas et al. [15] demonstrated that Arabidopsis seedlings ectopically expressing pea apyrase *psNTP9* exhibited increased apyrase-like activity in the cell wall fraction and imported Pi from eATP. In a recent study, the root [eATP] was inversely correlated with *AtAPY1* and *AtAPY2* expression levels [24].

Arabidopsis and soybean seedlings overexpressing the pea apyrase *psNTP9* also exhibited an unusual expansion of root system architecture (RSA) under conditions of P sufficiency [25]. Similar remodeling of RSA occurs in plants that are experiencing P starvation, increasing the root surface area for Pi scavenging from the soil [26,27]. Applied ATP is known to modulate root growth and development [28,29,30,31]. We hypothesize that apyrase overexpression in Arabidopsis seedlings may influence Pi acquisition both directly, via increased ecto-apyrase activities and release of Pi from NTP, and indirectly, by regulating eATP levels and purinergic signaling inputs into root development or other processes that influence Pi uptake. The major goal of this report was to experimentally test this hypothesis. Genome-wide expression profiling was employed to elucidate possible mechanisms by which changes in *APY1* expression may influence Pi acquisition in response to altered P availability and NTP supplementation in Arabidopsis seedlings.

## 2. Results

Preliminary experiments showed that after 10 days growth, there were no significant differences in seedling RSA or Pi contents on P-sufficient or P-limited media (Figure S1), suggesting that seedling P reserves remained sufficient to support normal growth until this stage of development. In seedlings growing on +P medium, lateral root (LR) development began to occur by day 12 with further expansion of RSA occurring by day 15. On −P medium, day 15 seedling Pi contents were two-fold lower, and expansion of RSA occurred to a greater extent than in seedlings grown on +P medium, which is a typical response to Pi limitation. The development of the LR system under P limitation occurred much later in the Ws ecotype than in Arabidopsis Col-0 lines [32], which are more widely used in studies of P metabolism.

We chose the day 15 stage of development to investigate: (1) whether acquisition of Pi via the metabolism of extracellular NTP could support seedling growth and development on −P medium and, potentially, increase P availability to seedlings growing on +P medium, and (2) the effects of *APY1* overexpression on these processes. In these experiments, NTP was continuously supplemented in the medium during the entire 15 days of growth.

### 2.1. Effects of P Limitation and Continuous NTP Supplementation on Seedling Root System Architecture and Pi Contents

**Figure 1:**
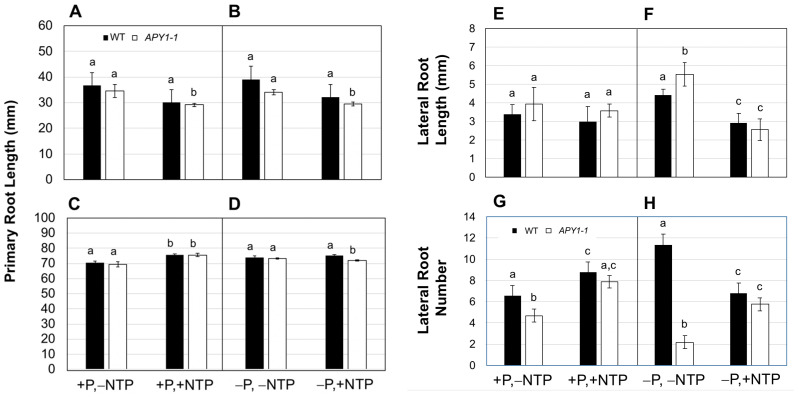
Changes in root system architecture of WT and *APY1-1* Arabidopsis seedlings grown on ±P medium, with or without NTP supplementation. Primary root lengths for day 10 (**A**,**B**) and day 15 (**C**,**D**) seedlings, showing transient inhibition of root growth by NTP on both P-sufficient (+P) and P-limited (−P) media. Effects of NTP supplementation (+NTP) on LR length (**E**,**F**) and number (**G**,**H**) in day 15 seedlings are shown. Data are means ± S.E. (*n* ≥ 10 seedlings) and are representative of two independent assays. Lowercase letters indicate significant differences, relative to non-NTP supplemented (−NTP) control treatments (+P or −P), as determined by one-way analysis of variance (1-ANOVA) with post hoc Tukey honest significant difference (HSD) testing (*p* ≤ 0.01).

At day 10, NTP supplementation resulted in a slight inhibition of primary root growth in both WT and *APY1-1* seedlings growing on either +P or −P medium (Figure 1A,B) but this change was only significant in *APY1-1* seedlings. At day 15, there were essentially no differences in primary root lengths (Figure 1C,D), perhaps due to more complete depletion of eNTP in the medium by this later time point. In +P medium, NTP supplementation did not alter the LR length (Figure 1E) but slightly increased the LR number (Figure 1G) in both WT and *APY1-1* seedlings. On −P medium, the RSA of WT seedlings was further expanded, mainly due to a two-fold increase in the LR number (Figure 1H). In contrast, *APY1-1* seedlings responded to P limitation with a two-fold decrease in the LR length (Figure 1F) and a three-fold increase in the LR number (Figure 1H). NTP supplementation prevented RSA remodeling under P limitation in both WT and *APY1-1* seedlings (Figure 1F,H), with growth and numbers of LR being similar to that seen in seedlings growing on +P medium without NTP supplementation. In seedlings grown on either ±P medium, the main effect of NTP was on LR initiation, rather than elongation.

**Figure 2:**
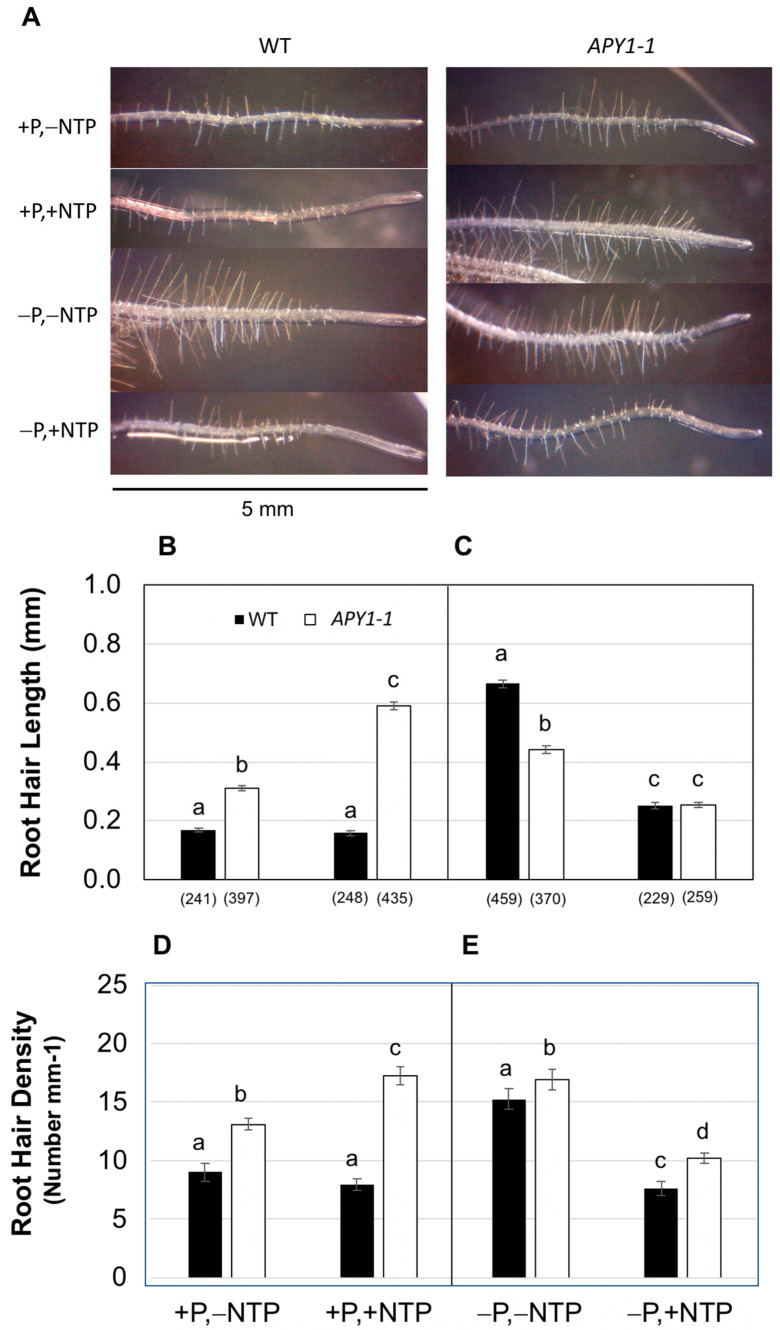
Root hair (RH) distribution in the distal 0.5 cm of root tips of 10-day-old Arabidopsis WT or *APY1-1* seedlings grown on medium with P (+P) or without P (−P), without NTP (−NTP) or with NTP (+NTP) supplementation (**A**). Effects of NTP on RH length in seedlings grown on +P medium (**B**) or −P medium (**C**), or RH density in the same seedlings grown on +P medium (**D**) or −P medium (**E**) are shown. The total number of RH measured in each sample are indicated in parentheses under the RH length graph. Lowercase letters indicate significant differences, relative to non-NTP supplemented +P or −P controls, as determined by 1-ANOVA with post hoc HSD testing (*p* ≤ 0.01).

As previously noted, overall RSA and seedling Pi contents were not different in day 10 WT seedlings growing on medium with or without P (Figure S1). However, increased root hair (RH) number and density in the −P seedlings indicated that these seedlings were, in fact, beginning to experience P limitation at this developmental stage (Figure 2; [33]). WT seedlings grown on −P medium supplemented with NTP exhibited decreased RH growth and density (Figure 2C,E). NTP did not enhance RH initiation or elongation in WT seedlings growing on +P medium (Figure 2B,D). In contrast, NTP increased both RH length and density in *APY1-1* seedlings growing on +P medium (Figure 2B,D). Even without NTP supplementation, RH growth and density were also higher in *APY1-1* roots relative to WT roots (Figure 2B,D). In response to P limitation, *APY1-1* seedlings exhibited only small increases in RH growth and density, and this response was inhibited by NTP supplementation of the medium (Figure 2B–E).

**Figure 3:**
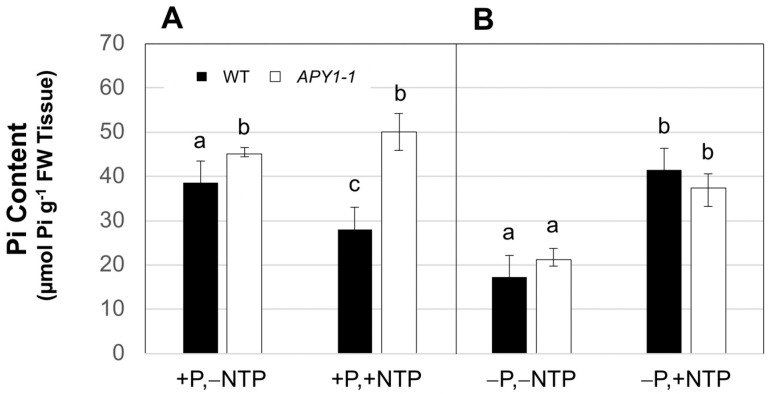
Effects of NTP supplementation on Pi contents of 15-day-old seedlings growing under P sufficiency (**A**) or limitation (**B**). Data are means ± S.E for four biological replicates of three to five seedlings each and are representative of two independent assays. Lowercase letters indicate significant differences, for NTP supplemented (+NTP) seedlings, relative to non-NTP supplemented (−NTP) control seedlings growing on P-sufficient (+P) or P-limited (−P) medium, as determined by 1-ANOVA with post hoc HSD testing (*p* ≤ 0.01).

On +P medium, the Pi contents of day 15 *APY1-1* seedlings were slightly higher than those of WT seedlings (Figure 3A). NTP supplementation of this medium did not significantly increase Pi contents of *APY1-1* seedlings but reproducibly decreased those of WT seedlings by approximately 25%. In comparison with *APY1-1*, the Pi content of these seedlings was nearly two-fold lower (Figure 3A). The Pi contents of both WT and *APY1-1* seedlings grown on −P medium were reduced two-fold (Figure 3B), relative to seedlings growing on +P medium, and NTP supplementation resulted in Pi contents that were not different from non-NTP supplemented +P controls (Figure 3A). Moreover, the Pi contents of the NTP supplemented WT and *APY1-1* seedlings on −P medium were also not different from each other at day 15 (Figure 3B).

### 2.2. Effects of P Limitation and Short-Term NTP or Pi Supplementation on Seedling Root System Architecture and Pi Contents

Continuous NTP supplementation of −P medium for 15 days did not result in increased Pi contents of *APY1-1* seedlings, compared with WT seedlings, as would be expected if higher ecto-APY1 activities played a significant role in Pi acquisition from eNTP. We therefore tested the possibility that effects of short-term NTP supplementation might reveal differences in Pi recruitment that were not seen in longer-term feeding experiments.

**Figure 4:**
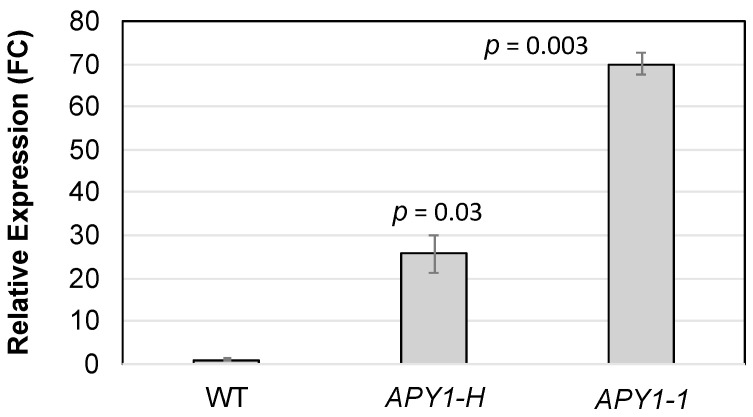
qRT-PCR analysis of *APY1* expression levels in two independent *APY1* overexpression lines, relative to that in WT seedlings (fold-change values, FC). Data are means ± SE for four biological replicates (three–five day seven seedlings), three technical replicates each. Significance values are from a Student’s *t*-test.

In these experiments, the day 10 seedlings growing on ±P medium were supplemented with NTP for two days. Photographs of representative seedlings are shown in Figure S2. These assays included two independent *APY1* overexpression lines, *APY1-1*, which was used in the previous 15-day assays, and *APY1-H*. The relative expression of *APY1* in each line, compared to WT, was quantified with qRT-PCR (Figure 4).

**Figure 5:**
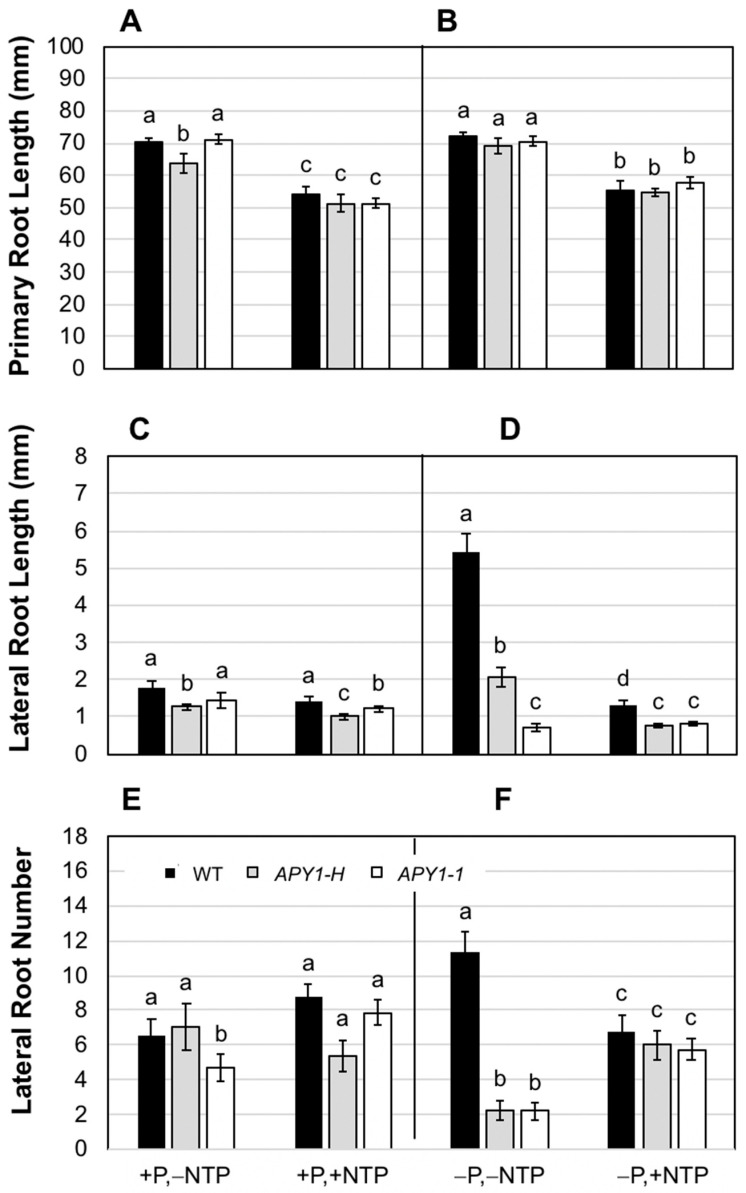
Changes in root system architecture of day 12 Arabidopsis seedlings in response to differences in P availability or supplementation with NTP for two days, beginning at day 10. Changes in primary root (**A**,**B**) and lateral root lengths (**C**,**D**) and number (**E**,**F**) per seedling were compared for WT versus two independent lines which overexpress *APY1* (*APY1-H*, *APY1-1*). Data are means ± S.E. (*n* ≥ 10 seedlings) and are representative of two independent experiments. Lowercase letters indicate significant differences for NTP supplemented (+NTP) seedlings, relative to non-NTP supplemented (−NTP) control seedlings growing on P-sufficient (+P) or P-limited (−P) medium, as determined by 1-ANOVA with post hoc HSD testing (*p* ≤ 0.01).

After two days of NTP supplementation of either +P or −P medium, both WT and *APY1* lines exhibited primary root shortening at day 12 (Figure 5A,B). NTP supplementation of +P medium produced only small changes in the LR length or number in all seedlings (Figure 5C,E). Under P limitation, the LR length and numbers increased two- to three-fold in WT seedlings but the most significant effect of this treatment in the *APY1* seedlings was a three-fold decease in the LR numbers (Figure 5E,F), as was previously noted for day 15 seedlings on −P medium (Figure 1C,G,H). These responses to P limitation were not seen when −P medium was supplemented with NTP (Figure 5D,F), consistent with the longer-term continuous NTP supplementation response. The LR system was similar to that of seedlings growing on +P medium, with or without NTP supplementation.

**Figure 6:**
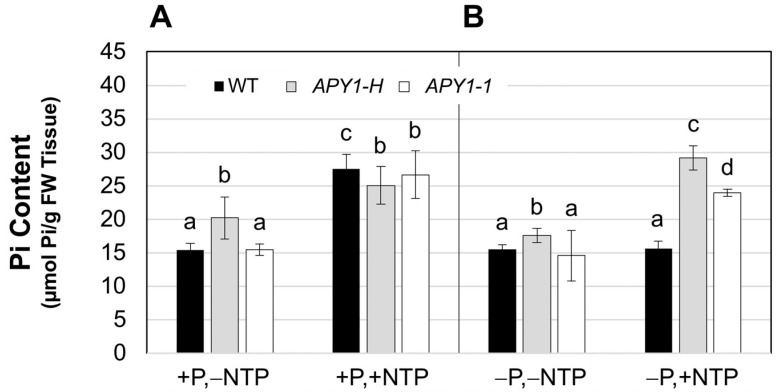
Effects of short-term NTP supplementation on Pi contents of day 12 seedlings growing under P sufficiency or P deficiency conditions. WT seedlings or two independent *APY1* overexpression lines (*APY1-H*, *APY1-1*) were grown on +P or −P medium for 10 days, then supplemented with NTP for two additional days. Data are means ± S.E. (*n* ≥ 10 seedlings) and are representative of two independent experiments. Lowercase letters indicate significant differences for NTP supplemented (+NTP) seedlings, relative to non-NTP supplemented (−NTP) control seedlings growing on P-sufficient (+P) or P-limited (−P) medium, as determined by 1-ANOVA with post hoc HSD testing (*p* ≤ 0.01).

The Pi contents of WT and *APY1-1* seedlings were similar on +P medium, and NTP supplementation increased their Pi contents by approximately two-fold (Figure 6A). The significantly higher Pi contents of *APY1-H* seedlings on +P, −NTP medium were not further increased after two days of NTP supplementation (Figure 6A). At day 12, the Pi contents of seedlings growing on −P medium were still nearly identical to those of seedlings on Pi medium (Figure 6B), suggesting that most of the reduction in tissue Pi contents observed at day 15 occurred between days 12 and 15. NTP supplementation did not increase Pi contents of WT seedlings, while those of *APY1* seedlings increased two-fold (Figure 6B).

### 2.3. Genome-Wide Expression Profiling to Investigate Responses of WT and APY1 Seedlings to P Limitation and NTP Supplementation

We performed pairwise comparisons of gene expression profiles in day 15 WT and *APY1-1* seedlings to identify differentially expressed genes (DEG) across the four experimental treatments. In total, 3781 unique genes were DE in one or more comparison groups. qRT-PCR validation for three DEG in the RNA-seq datasets is provided in Table S1. Fold-change expression values and annotation data were arrayed in a single matrix for comparison of expression data for individual genes, by treatment and by genotype. Sets of DEGs were also subjected to Venn analyses comparing responses of the individual WT or *APY1-1* seedlings to different experimental treatments or comparing WT and *APY1* seedling responses under the same treatments. In each overlap analysis, sets of genes shared by each set of DEG from a pairwise comparison, and sets of DEG unique to one or the other comparison group, were analyzed for GO BioProcess category enrichment. Genes and annotations for highly overrepresented categories were retrieved. This approach facilitated interpretation of the major differences and similarities between WT and *APY1-1* seedling transcriptomic responses to experimental treatments.

As noted previously, most studies of P metabolism have been conducted using Arabidopsis Col-0 accessions, rather than the Ws ecotype used in the present study. Therefore, we first examined gene expression changes in WT and *APY1-1* seedlings under Pi limitation to assess their responses compared with well-characterized Col-0 responses. We then investigated differences in gene expression between WT and *APY1-1* seedlings, related to their capacity to recover Pi from exogenous NTP in both P-replete and P-deficient media.

#### 2.3.1. Gene Expression Changes in Response to P Limitation

Expression profiles for PSR genes in both WT and *APY1-1* seedlings were very similar to those in Col-0 seedlings [34]. Among the up-regulated DEG shared by WT and *APY1-1* seedlings, many are involved in Pi homeostasis or cellular responses to P starvation, and sulfolipid or galactolipid biosynthesis (Figure S3A; Table S2). Catabolism of phospholipids and their replacement with non-phosphorous galactolipids and sulfolipids is an adaptation to P limitation [34]. Additional genes encoding Pi transporters or enzymes of flavonoid metabolism were enriched only in *APY1-1* seedlings (Figure S3A; Tables S2 and S3).

In both WT and *APY1-1* seedlings, growth on Pi-deficient media induced genes involved in the sensing of cellular Pi levels and regulation of Pi homeostasis (*ITPK2*, *IPS1*, *IPS2/At4*, *SPX3*; Table S2). Up-regulation of genes encoding secreted purple acid phosphatases and the major inorganic Pi transporters (PHT1;1, PHT1;2, PHT1;4) would enhance extracellular Pi scavenging and uptake [2,9,35,36,37]. In *APY1-1* seedlings, stronger induction of PHT1;2 and induction of additional genes encoding several acid phosphatases, Pi transporters (PHT1;5, PHT1;9, PHO1;H1), and PHF1, a protein that promotes Pi uptake by facilitating trafficking of PHT1 transporters to the plasma membrane [38] are consistent with an increased Pi uptake potential. *APY1-1* seedlings also exhibited increased expression of genes for SPX1 and SPX2 proteins, which communicate changes in the cellular Pi status, regulating cellular Pi homeostasis and expression of PSR genes by PHOSPHATE RESPONSE 1 (PHR1) and other core elements of the Pi regulon [39,40]. Interestingly, expression of *APY1* and other apyrases (*APY2-7*) was not significantly different in response to P limitation in either WT or *APY1-1* seedlings, suggesting that apyrases themselves are not PSR genes.

Accumulation of anthocyanins is a well-documented response to long-term P starvation in plants [3]. In *APY1-1* seedlings, expression of genes encoding key enzymes regulating the synthesis of flavonones (CHS, CHI3), dihydroflavonols (F3H), and flavonols (FLS1), as well as UDP-glycosyltransferases required for anthocyanin accumulation was increased by this treatment (Table S3; [41]). In contrast, *CHI* and *FLS1* were only weakly induced in WT seedlings experiencing Pi deprivation. However, anthocyanidin synthesis genes (*DFR*, *ANS*) were not DE, and increased anthocyanin levels were not detected in either WT or *APY1-1* seedlings, relative to those grown on +P medium.

Genes regulating root development were also enriched in both WT and *APY1-1* seedlings under P limitation (Figure S3A), consistent with previous studies that have characterized an expanded RSA as a hallmark of P starvation [26]. These included genes encoding peroxidases and other cell wall modifying enzymes (PER44, PER73, XTH14), nitrate transporter NRT2.1 and PSR and auxin-inducible genes that regulate LR and RH development (Table S4).

#### 2.3.2. Transcriptome Responses to NTP Under P Limitation

In −P medium, relatively small numbers of genes were DE between WT and *APY1-1* seedlings, with or without NTP supplementation (Table S2) and both lines responded to P limitation in a similar manner (Section 2.3.1). However, the phosphate starvation response was more pronounced in *APY1-1* seedlings than in WT seedlings (Table S2), with a higher relative expression of genes for phospholipid remodeling (*MGD3*, *SQD1*, *SQD2*, *GPDP1*, *PLPZETA2*), Pi mobilization (*PPA1*, *PPA2*, *PPA4*, *PAP14*), uptake (*PHT1*;*4*), and regulation of Pi homeostasis (*SPX1*, *SPX2*). When NTP was supplied as the sole source of P, a small number of these PSR genes remained up-regulated in day 15 WT seedlings, even though their Pi contents were not significantly different from those of WT seedlings and were only slightly lower than in WT or *APY1-1* seedlings grown on +P medium without NTP supplementation.

Under P limitation, neither WT or *APY1-1* seedlings exhibited differences in the expression of flavonoid biosynthesis genes, with or without NTP (Table S3). Expansion of RSA in WT and *APY1-1* seedlings growing on −P medium was largely prevented by NTP supplementation, and there were relatively few differences in expression of genes associated with root development or auxin responses (Figure 1, Figure 2 and Figure S4; Tables S4 and S5).

#### 2.3.3. Transcriptome Responses to NTP Under Conditions of P Sufficiency

Under conditions of P sufficiency, relatively few genes were DE in response to NTP in WT seedlings (Figure S3B). In contrast, NTP supplementation of +P media influenced the expression of large number of genes in *APY1-1* seedlings, generally repressing genes involved in water deprivation or abscisic acid responses (Figure S3B). These included genes encoding enzymes of cuticular wax synthesis and plasma membrane and tonoplast aquaporins. As has previously been reported [42], genes involved in indole glucosinolate biosynthesis were induced by NTP, likely due to increased *HIGH INDOLE GLUCOSINOLATE 1* (*HIG1/MYB51*) expression [43]. The latter response was not observed under P limitation (Figure S3A), where induction of sulfolipid synthesis genes, related to phospholipid remodeling, paralleled repression of genes for synthesis of S-containing glucosinolates.

In +P medium without NTP, few genes regulating P acquisition, Pi transport, or regulation of Pi homeostasis were DE between WT or *APY1-1* seedlings (Figure S3B; Table S2) and do not offer a possible explanation for the slightly elevated Pi contents of *APY1-1* seedlings (Figure 3). *APY1-1* roots did exhibit an increased RH length and density (Figure 2B,D), compared with WT seedlings, and expression of a number of genes regulating RH initiation and growth were induced in *APY1-1*, relative to WT seedlings (Table S5). Other RSA characteristics were not significantly different, and genes regulating root development were not enriched in *APY1-1* seedlings (Figure S3B).

The Pi content of *APY1-1* seedlings grown on +P medium supplemented with NTP was nearly two-fold higher than that of WT plants, resulting from both an increase in Pi content in *APY1-1* seedlings and a decrease in WT seedlings. As is seen in Table S2, the lower relative expression of a large number of genes involved in P acquisition and homeostasis regulation likely reflects an adjustment to the higher tissue Pi contents in *APY1-1* seedlings at day 15. Significantly increased LR number and RH length and density in these seedlings likely facilitated increased Pi uptake. Compared with *APY1-1* seedlings, only three PSR genes were DE in NTP supplemented WT seedlings growing on +P medium (Table S2) and only a small increase in LR number was observed. Thus, gene differential expression data do not fully explain the unexpected decrease in Pi contents of these seedlings in response to NTP supplementation. This result in day 15 seedlings was observed in two independent trials, although Pi contents of WT seedlings grown on +P medium ±NTP were not different at day 10 (Figure S1), or after short-term (2 days) NTP supplementation (Figure 5A). Interestingly, apyrase 5 (*APY5*) expression was five-fold higher in *APY1-1* seedlings than in WT seedlings, in response to NTP. This was not observed in seedlings grown on +P medium without NTP, suggesting that induction of *AtAPY5* is NTP-dependent, at least under P sufficiency conditions, as it was not detected under P limitation. It remains unclear whether *APY5* functions as an ecto-apyrase or contributes to enhanced Pi acquisition from NTP in *APY1-1* seedlings.

On +P media, NTP strongly repressed a large number of flavonoid biosynthesis genes in *APY1-1* seedlings. This response was attenuated in WT seedlings and absent in NTP supplemented seedlings growing under P limitation (Table S3).

The increased LR number (Figure 1) and RH length and density (Figure 2) in *APY1-1* seedlings on +P, +NTP medium was accompanied by a very large number of DEG involved in auxin responses (Table S4), consistent with the well-established role of auxin in regulating root growth [44].

In *APY1-1* seedlings growing on +P medium, genes regulating flavonoid synthesis were down-regulated, relative to WT. NTP supplementation of this medium further repressed additional genes involved in flavonoid synthesis and transport in *APY1-1* seedlings (Table S3), resembling their response to P limitation. To our knowledge, NTP regulation of flavonoid synthesis has not been previously reported.

Supplementation of the +P medium with NTP also resulted in enrichment of a large number of genes involved in phytohormone responses in *APY1-1* but not in WT seedlings. More than 100 genes regulating auxin signaling, homeostasis, and responses were DE (Table S4). Many of these genes are known to regulate root development and likely contributed to the unexpected increase in LR numbers (Figure 1G) under P sufficiency conditions. These included the transcription factor UPBEAT1, which regulates the expression of a set of peroxidases that modulate the balance of reactive oxygen species (ROS) between the zones of cell proliferation and the zone of cell elongation where differentiation begins in the root [45] and MYB73/77 and NAC001, which regulate LR development [46,47]. Numerous up-regulated SAURs and cell wall remodeling enzymes (XTH22, pectin lyases, expansins) function in cell expansion growth.

A shared response to NTP in both WT and *APY1-1* seedlings in +P medium was the induction of *PHO2*. PHO2 (ubiquitin-conjugating enzyme E2 targets PHT1 and PHO1 transporters for turnover [48], as would be expected under conditions of increased P availability. PHO2 is essential for the maintenance of phosphate homeostasis, preventing excess Pi accumulation and toxicity [49].

**Figure 7:**
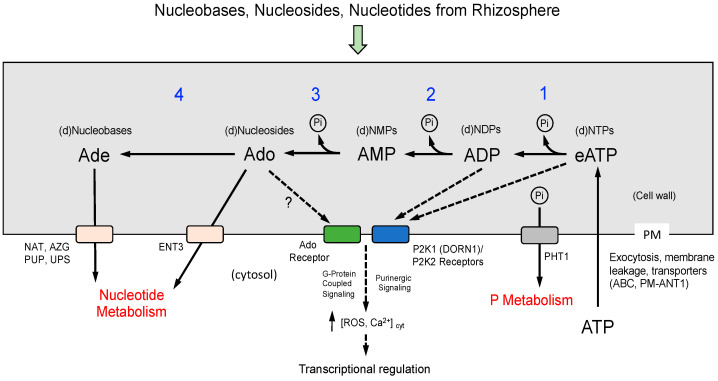
Model for apoplastic salvaging of extracellular ATP (eATP) and potential signaling pathways involving purinergic receptors or Ado receptors. Cellular and extracellular sources of eATP (and other nucleotides) are indicated. Direct uptake of nucleotides via plasma membrane NTP transporters is not known to occur. Metabolism of eATP to AMP (steps 1, 2) may occur via ecto-apyrase activities, with further metabolism of AMP to adenosine (Ade) by a 5′-nucleotidase activity (step 3), as was reported by [22]. Non-specific ec-to-phosphatase activities, such as purple acid phosphatases, may also metabolize eATP (steps 1, 2, 3; [18]. Pi uptake is facilitated by PM-localized PHT1 inorganic phosphate transporters. PM-localized equilibrative nucleoside transporter 3 (ENT3) imports Ado or other nucleosides into the cell [52]. In potato and Arabidopsis, most Ado is further metabolized to adenine (Ade) by the cell wall-localized, purine-specific nucleosidase NSH3 (step 4; [22,53]). Ade is then imported into the cell, potentially by one or more PM-localized nucleobase transporters [54]. Imported nucleosides and nucleobases may then enter salvaging pathways for intracellular nucleotide synthesis, or be catabolized to release nitrogen to general N metabolism [51,54]. Apoplastic salvaging reactions would also regulate levels of eATP, an important signaling molecule which binds P2K1- and P2K2-type purinergic receptors, initiating intracellular signaling cascades that regulate a variety of growth processes and defense responses in plants [13]. These receptors also bind ADP with high-affinity [55,56]. Little is known about plant Ado receptors [57] or how Ado-mediated signaling is coordinated with eATP signaling. Increased intracellular concentrations of ROS and Ca^2+^ initiate signaling cascades resulting in transcriptional regulation of genes.

Gene expression data also provided insights into potential mechanisms of the uptake and metabolism of nucleosides and nucleobases derived from NTP salvaging in the apoplast (see model, Figure 7). In *APY1-1* seedlings, NTP supplementation of P-sufficient medium induced PM-localized purine permeases PUP14 and PUP18, which function in the uptake of extracellular salvage pathway products such as adenine and cytosine [50], whereas genes encoding enzymes of intracellular de novo synthesis or salvaging pathways were repressed, and catabolic pathway genes were induced (Table S6). These metabolic responses characterize pyrimidine salvaging in Arabidopsis seedlings [51].

Beyond serving as a Pi source, extracellular NTP or nucleoside products of NTP salvaging may also initiate signaling cascades that alter gene expression and seedling development (Figure 7). Differences in eNTP metabolism between WT and *APY1-1* seedlings could modulate these responses.

## 3. Discussion

Transcriptome-wide PSR gene expression changes in Arabidopsis WT and *APY1-1* seedlings were similar to those described for the more widely used Col-0 accession [34]. One notable difference was the lack of significant anthocyanin accumulation under P limitation, which has been linked to a phytochrome D mutation (*phyD-1*) in the Ws ecotype [58]. In salt-stressed Arabidopsis seedlings, Leschevin et al. [59] reported significantly lower levels of anthocyanidin synthase protein in Ws seedlings, compared with Col-0 seedlings, and neither *DFR* or *ANS* were induced by Pi limitation in WT or *APY1-1* seedlings in the present study.

WT seedlings also exhibited a markedly different RSA, compared with Col-0 seedlings [32]. In Col-0 seedlings growing on P-replete medium, extensive LR development was seen by day 10, when the first LR were initiated in WT seedlings. In response to P limitation, inhibition of primary root growth and further expansion of the LR system occurred by day 10 in Col-0 seedlings [32]. In contrast, primary root growth inhibition was not observed in WT seedlings, and only limited LR system expansion began around day 10. The expanded RSA phenotype in both WT and Col-0 seedlings under P limitation resembles the auxin-induced response under P sufficiency. However, Williamson et al. [32] reported that RSA remodeling in response to P limitation was not different between Col-0 and several auxin-resistant mutants in the same background. In the present study, relatively few genes involved in auxin responses were DE in either WT or *APY1-1* seedlings grown on -P medium, consistent with previous findings. However, DE of a large number of auxin response genes and induction of LR and RH initiation by NTP under P sufficiency conditions in *APY1-1* but not WT seedlings, suggests that differences in NTP metabolism may regulate RSA remodeling via changes in auxin metabolism, transport, or responses.

### 3.1. Metabolism of eNTP as a Source of Pi in Arabidopsis Seedlings

A key question addressed by this study was whether enhanced *APY1-1* expression enables plants to more efficiently utilize NTPs as a source of Pi. A model for apoplastic NTP salvaging, Pi mobilization, and the role for apyrase in this process is shown in Figure 7. Some APY1 in Arabidopsis is localized in the Golgi, where it functions as an NDPase [60,61], but some is also localized in the extracellular matrix, functioning as an “ecto-apyrase” that regulates eATP levels [24,36,37,62,63]. Thus, over-expression of *APY1* and resulting elevated ecto-apyrase activities might enhance Pi acquisition from eNTP in the rhizosphere, similar to how apyrase contributes to extracellular nucleobase salvaging [22].

Although apyrase activities in extracellular matrix (ECM) fractions were not directly measured, the high *APY1* expression levels in *APY1-1* and *APY1-H* seedlings likely correspond to elevated apyrase activities in the ECM, as previously shown by Clark et al. [24] in Arabidopsis roots, and in an Arabidopsis line ectopically expressing *psNTP9*, the pea ortholog of *APY1* [15]. It remains unclear, however, to what extent elevated APY1 activity directly contributes to Pi mobilization from NTP. Pi-starvation inducible secreted purple acid phosphatases that function in Pi scavenging from a wide variety of organophosphates typically exhibit K_m_ values of 1–10 mM for NTP and NDP substrates [64,65]. In contrast, Arabidopsis APY1 exhibited a K_m_ = 30 µM for ATP [66] and 60–170 µM for NDPs [61], suggesting that it would be more effective in utilizing these substrates as a source of Pi. Thus, apyrases might be especially important in recruitment of Pi from NTP in soils, where NTPs may be present in micromolar concentrations. Native Arabidopsis apyrase genes do not appear to be Pi-starvation responsive. NTP supplementation also did not change expression of *APY* genes, with the notable exception of *APY5*, which was strongly induced by NTP in *APY1-1* seedlings grown in +P medium. This increase may have contributed to the elevated Pi contents of these seedlings.

In Pi-sufficient medium, NTP supplementation had no significant effects on WT seedling growth and development or gene expression in day 15 seedlings and, unexpectedly, their Pi contents were slightly reduced. The reasons are unclear, since the LR numbers were slightly increased and RH density and length remained unchanged in response to NTP. In contrast, the same treatment produced profound changes in day 15 *APY1-1* seedlings. In addition to having Pi contents that were nearly two-fold higher than in WT seedlings, which may have resulted from an increased release of Pi from NTP, the increased surface area provided by their increased LR number and RH density and length would facilitate Pi uptake from the medium. High-affinity PHT1 transporters are expressed primarily in the root epidermis and RH [27], thus the enhancement of RH development by NTP is an important feature of RSA remodeling and Pi acquisition in the *APY1-1* seedlings. Because low [eATP] promotes RH growth and high [eATP] inhibits it [28], elevated ecto-APY1 activities would help maintain lower [eATP], thereby promoting RH growth. Consistent with this, functional complementation of the rice *root hairless 1* (*rth1*) mutant with *OsAPY1* cDNA restored normal RH development [67].

Interestingly, the decreased Pi content observed in day 15 WT seedlings grown on +P medium with continuous NTP supplementation was not seen in seedlings grown on +P medium for 10 days, followed by two days of NTP treatment. Pi contents in WT and two independent *APY1* lines were similar to each other at day 12, each being approximately two-fold higher than at day 10. This suggests that both genotypes had comparable Pi mobilization and uptake capacities and that NTP can serve a significant Pi source even under P sufficiency. Because RSA was similar at the start of treatment (day 10), the shorter NTP supplementation period was likely inadequate to drive major RSA remodeling beyond slight inhibition of primary root growth (Figure 4). The potential for higher ecto-APY1 activities in *APY1* seedlings to mobilize Pi from eNTP may also have been offset by the decreased expression of numerous genes encoding other phosphatases and Pi transporters, which was seen in response to NTP supplementation of +P medium.

In Pi-deficient medium, an ecto-apyrase-enhanced recovery of Pi from eNTPs would be expected to increase Pi content in *APY1-1* seedlings, relative to WT, but this was not observed after 15 days of continuous supplementation with NTP. Pi contents and RSA were similar between genotypes, suggesting comparable Pi uptake capacities. We did not measure Pi uptake directly, but Thomas et al. [15] demonstrated that identically grown day 15 Arabidopsis seedlings ectopically expressing pea apyrase *psNTP9* exhibited a significantly higher Pi uptake than WT seedlings after transfer from low-Pi (0.1 mM Pi) to high-Pi (2 mM Pi) medium. Thus, the *psNTP9* line had a more robust phosphate starvation response, characterized by enhanced Pi uptake when Pi availability increased. We also observed this after short-term NTP supplementation times in the present study. Increased Pi contents by both *APY1* lines, but not WT seedlings, occurred after two days of NTP supplementation on −P medium, supporting an enhanced acquisition of Pi from NTP as a sole source of P, even though this was not apparent after 15 days of continuous supplementation. Since the day 10 seedlings grown on ±P medium did not exhibit differences in RSA or Pi contents at the time of NTP supplementation, one possible explanation for the differences in Pi scavenging is that NTP inhibited RH development in WT but not APY1 seedlings. It seems likely that the uptake of Pi mobilized from NTP occurred at a faster rate initially in *APY1* seedlings than in WT seedlings, with both acquiring approximately the same total amounts of Pi over 15 days, as NTP levels in the medium were exhausted, decreasing inhibition of RH development in WT seedlings.

Apoplastic NTP salvaging could also impact the uptake and intracellular metabolism of nucleosides and nucleobases. Consistent with this prediction, NTP supplementation of *APY1-1* seedlings in +P medium induced genes for transporters and enzymes that facilitate uptake and catabolism of the nucleobase products of apoplastic NTP salvaging. Induction of catabolic pathway activities would increase remobilization of N from purines and pyrimidines into general N metabolism [51,54,68]. Thus, complete salvaging of nucleotides would enhance both P and N availability in plants.

### 3.2. Extracellular NTP Signaling in APY1 Seedlings Growing Under Pi-Sufficiency

While most previous studies have examined the role of eATP in plant growth and development or stress responses, this study focused on Pi acquisition from eNTP, so a mixture of pyrimidine and purine nucleotides was provided to the seedlings to better represent the situation at a natural root/soil interface. Differential responses to NTP by WT and *APY1* seedlings may reflect differences in apoplastic eATP metabolism and purinergic signaling, as well as complex signaling mechanisms involving receptors for other extracellular nucleotides [13] or their metabolites, such as adenosine ([57]; see Figure 7).

Phosphate limitation dampens the downstream free Ca^2+^ signature component of purinergic signaling, which is induced at the root tip by eATP and modulates transcriptional responses [69]. Consistent with this observation, we found few significant differences in WT or *APY1* DEGs or phenotypes in response to continuous NTP supplementation of seedlings grown on −P medium. The very different Pi mobilization response to short-term NTP feeding probably results from the fact that the day 10 seedlings growing on either ±P media had similar Pi contents at the time of NTP supplementation. Reduced Pi acquisition by WT seedlings in this experiment may be explained by the inhibitory effects of millimolar NTP on purinergic signaling, relative to *APY1* lines, since [eATP] has been shown to be inversely proportional to *AtAPY1* and *AtAPY2* expression in Arabidopsis roots [24].

Previous studies have shown that eATP influences root growth and development, and several lines of evidence suggest that ecto-apyrase regulation of [eATP] and purinergic signaling impacts polar auxin transport, which is required for LR and RH formation [44]. High *APY1* expression promoted shoot-to-root auxin transport and LR formation in Arabidopsis seedlings, while repression of *APY1* expression had the opposite effect [30,62]. Applied high concentrations of eATP, which is analogous to the situation in which *APY1* expression is low, increased auxin sensitivity in root tissues and disrupted basipetal auxin transport out of primary root tips, promoting LR formation as a result of auxin accumulation in root tissues [29]. These and other studies suggest that AtAPY1 regulates root growth and development by controlling [eATP], with altered auxin transport being one downstream response. It is noteworthy that the expression of these apyrases in light-grown seedlings is highest in root tip tissues where PIN auxin efflux carriers are also highly expressed [62]. *APY1* expression is normally restricted to growing cells, and it is unclear to what extent constitutive expression of *APY1* throughout the plant contributed to enhanced of auxin transport and expanded RSA phenotype observed upon NTP supplementation in the present study.

Based on these findings, NTP supplementation of WT seedlings growing on +P medium would be expected to decrease basipetal auxin transport into primary roots. This change would reduce primary root growth and auxin-regulated LR formation, which was observed. However, transcriptome data showed that few auxin response genes were DE in these seedlings, suggesting that this response to NTP was not primarily auxin-regulated.

Conversely, increased ecto-apyrase activities in *APY1* seedlings grown on +P medium would be expected to reduce [eNTP], thus increasing basipetal auxin transport into the roots and promoting LR formation, which was also observed. In *APY1* seedlings, large numbers of genes involved in auxin-regulated growth were induced on +P, +NTP medium. Notably, the coordinated down-regulation of genes for the synthesis of flavonoids, which include polar auxin transport inhibitors like quercetin [70], and increased expression of polar auxin transport components (AUX1, PIN4, PIN7) are consistent with enhanced auxin transport into the root. In the root tip, these auxin transporters play major roles in re-directing auxin flow (basipetal transport) into pericycle tissues, the site of LR initiation. They also help maintain auxin gradients that regulate root growth [71]. These findings, supporting earlier work, help to explain how the overexpression of *APY1* may lead to an expanded RSA for Pi acquisition, further increasing the Pi contents of these seedlings under non-P-limiting conditions. A similar role for auxin in RSA remodeling has been proposed for Arabidopsis seedlings, which are ectopically overexpressing *psNTP9* [25].

NTP supplementation of *APY1* seedlings growing under P limitation did not result in changes in the expression of genes that regulate auxin-induced growth, and LR formation was suppressed in both WT and *APY1* seedlings. Expansion of RSA under P limitation results partly from increased auxin sensitivity in root tissues [44], thus the stronger inhibition of LR formation in *APY1* plants supplemented with NTP may result from the accumulation of inhibitory levels of auxin in root tissues.

We have shown that, under Pi deficiency, both WT and *APY1* plants can efficiently recruit Pi from NTP as a sole source of P, supporting normal seedling growth and development. In native soils, in which NTP are likely to be present at micromolar concentrations, the kinetic properties of apyrase may favor it over other ecto-phosphatases in the mobilization of Pi from NTP, contributing to overall P nutrition in plants. An unexpected, but important, finding of this study is that, relative to WT, the Pi contents of *APY1* seedlings were also increased under conditions of Pi-sufficiency, both in the presence and absence of NTP. A similar enhancement of Pi uptake in several different plants that ectopically express the pea *psNTP9* ortholog of *APY1* [72] supports this finding.

## 4. Materials and Methods

### 4.1. Growth of Arabidopsis Seedlings

*Arabidopsis thaliana* ecotype Wassilewskija (Ws-2) wild-type and two APY1 overexpression lines in the Ws background (*APY1-H*, *APY1-1*) were used in the present study. Seeds were surface sterilized and plated on 1/2x Murashige–Skoog (MS) medium with phosphate (0.612 mM Pi) or without Pi (Plant Cell Technology, Smithfield, UT, USA), 0.5% (*w*/*v*) MES, adjusted to pH 5.8 with KOH. Solid medium was 0.5% [*w*/*v*] Gelzan CM (Millipore-Sigma, St. Louis, MO, USA). Some plates were supplemented with sterile-filtered 1.25 mM mixed nucleotides (1:1:1:2 ATP, GTP, CTP, UTP; 0.25 mM each for ATP, GTP, CTP, and 0.5 mM UTP), prepared according to the manufacturer’s recommendations (Millipore-Sigma, St. Louis, MO, USA). For root growth assays, plates were stratified for 2 days at 4 °C, then vertical plates (15–20 seeds per plate) were grown at 22 °C under a 16L:8D photoperiod and full-spectrum LED grow lights (approximately 150 μmoles m^−2^ s^−1^ Photosynthetic Photon Flux Density, PPFD). Plants grown for seed stocks were planted in a standard potting mix (BM2 seed germination mix; Berger, Saint-Modeste, QC, Canada) and maintained in a growth chamber under 50–70% humidity and identical temperature, photoperiod, and light intensity as plated seedlings.

### 4.2. Root System Architecture (RSA) Analyses

ImageJ v 1.48 (https://imagej.net/ij/, accessed on 3 December 2025) software was used to analyze root images of vertically grown seedlings. Primary and lateral root lengths were measured for *n* ≥ 10 seedlings and root hair (RH) number and length were measured for the distal 0.5 cm segment of primary root tips.

### 4.3. Seedling Phosphate Content Assay

Seedlings were harvested from plates, rinsed in dH_2_O, blotted, and weighed. Tissues were homogenized in 1 mL of 1N H_2_SO_4_ and extracts were incubated at 42 °C overnight in capped tubes. After centrifugation (16,000× *g*, 5 min), supernatant Pi contents were quantified using a modified assay described by [73]. Briefly, 10 µL of the hydrolysate and 90 µL dH_2_O was vortexed with 0.9 mL of freshly prepared Pi Color Solution (0.35% (*w*/*v*) ammonium molybdate, 1.4% (*w*/*v*) ascorbic acid in 1 N H_2_SO_4_. Following a 42 °C, 60 min incubation in the dark, A_820_ was measured for samples and Pi standards (1–300 nmoles KH_2_PO_4_). Pi contents were calculated from the Pi standard curve and expressed as µmol Pi g fresh weight^−1^.

### 4.4. Construction and Characterization of the APY1 Overexpression Lines

The full-length *AtAPY1* cDNA coding region (GenBank accession no. AF093604; [66]) was cloned into Gateway cloning vector pH7WG2 downstream of the Cauliflower mosaic virus 35S promoter. *Agrobacterium*-mediated plant transformation of *Arabidopsis thaliana* ecotype Wassilewskija (Ws-2) and hygromycin selection of transformants was as previously described [74]. *APY1* expression in independent transgenic lines was quantified by qRT-PCR using *APY1*-specific primers (Table S7). Verification of the Ws-2 genetic background in all lines was carried out by SNP genotyping of the AT5G42320 locus, as described by [75] using primers AT5G42320-Ws2-F, AT5G42320-Col0-F, and AT5G42320-Rev (Table S7).

### 4.5. RNA Isolation, cDNA Synthesis, and qRT-PCR Analyses

Total RNA was extracted from rapid-frozen seedling tissues using an RNeasy Plant Mini Kit, as per the manufacturer’s protocol (Qiagen, Valencia, CA, USA). RNA samples were treated using a TURBO DNA-Free DNase I kit (ThermoFisher Scientific, Waltham, MA, USA) to remove genomic DNA contamination prior to qRT-PCR or RNA-seq analyses. For qRT-PCR assays, RNA was reverse-transcribed using a SuperScript II kit (ThermoFisher Scientific, Carlsbad, CA, USA). qRT-PCR primers (Table S7) were designed using NCBI Primer-BLAST (www.ncbi.nlm.nih.gov/tools/primer-blast/, accessed on 3 December 2025) [76] so that one primer of each pair spanned an exon–exon junction, preventing amplification of gDNA. Reactions (20 µL) contained 5 μL of cDNA (1 ng/µL), 0.4 µL of each primer (10 μM), 10 μL of Power SYBR Green master mix and 4.2 μL nuclease-free water. qRT-PCR was conducted using a ViiA7 Real –Time PCR System (Applied Biosystems, ThermoFisher Scientific, Waltham, MA, USA) as follows: 95 °C for 10 min, followed by 40 cycles of 95 °C for 30 s, 58 °C for 30 s, and 72 °C for 30 s in 96-well optical reaction plates. Expression of reference gene *PP2A* (AT1G69960) was used to normalize target gene expression. Relative expression was calculated using the DDCT method [77]. Dissociation curve analyses were used to check for the amplification of homogenous products, whose size was verified using agarose gel electrophoresis, following secondary PCR amplification.

### 4.6. Genome-Wide Expression Analyses

Total RNA was extracted from 15-day-old seedlings that were collected 6 h after the beginning of the light period and treated with DNase I, as described above. cDNA library preparation and Illumina (San Diego, CA, USA) sequencing (NovaSeq 6000 SR100 platform; 100 bp single-end reads) using the Tag-seq method [78] was carried out by the Genome Sequencing and Analysis Facility (GSAF) at the University of Texas at Austin. Reads mapping and sample quality control analyses were performed as described by [25] for three biological replicates each of WT and *APY1-1* overexpression lines grown under four different experimental conditions (±P, ±NTP). An average of 13.4 million reads were mapped to the reference genome (TAIR 10.1, Araport 11 annotation; [79]) for each sample, with 81.0% mapping to genes (74.0% mapped to exons). Gene differential expression analyses were carried out using the R Bioconductor module DESeq2 [80]. Differentially expressed genes (DEG) were defined as having fold-change values ≥ 1.5, up or down, relative to WT expression, and *padj* ≤ 0.05. Comparison of DEG sets by Venn diagram analyses employed the web tool InteractiVenn (www.interactivenn.net/, accessed on 3 December 2025). Lists of DEG were analyzed for statistically over-represented gene sets in Biological Process Gene Ontology (GO) categories, using PANTHER classification system v 19.0 (www.pantherdb.org, accessed on 3 December 2025). Annotated gene sets for GO terms overrepresented by ≥two-fold (FDR *p* ≤ 0.05, Fisher test) were retrieved for further analyses.

### 4.7. Statistical Analyses

Statistical significance of *APY1* responses to different treatments, relative to WT responses, was assessed using Student’s *t*-test or 1-ANOVA with post hoc Tukey honest significant difference (HSD) testing or Student’s *t*-test (2-tailed, unequal variances).

## 5. Conclusions

Genome-wide expression profiling in the present study extends our understanding of the results of previous studies and supports a role for APY1 in Pi acquisition directly via metabolism of eNTP and by modulation of eNTP signaling and root development, which is essential for Pi uptake. These findings have potential application to the development of crops with enhanced fertilizer use efficiency and yields. Under field conditions, with periods of both limited Pi and increased Pi availability, following fertilizer applications, transgenic crops overexpressing *AtAPY1* would be expected to scavenge more Pi, potentially increasing phosphate use efficiency and limiting environmental degradation due to P leaching from soils.

## Data Availability

RNA-seq reads data have been deposited in the NCBI Sequence Read Archive (BioProject accession #PRJNA886722).

## Supplementary Materials

The following supporting information can be downloaded at: https://www.mdpi.com/article/10.3390/ijms262411857/s1.

## Abbreviations

The following abbreviations are used in this manuscript:

eATP	extracellular ATP
AMP	adenosine-5′-monophosphate
RSA	root system architecture
PSR	phosphate starvation response
LR	lateral roots
RH	root hairs
DEG	differentially expressed gene(s)
Pi	inorganic phosphate
ECM	extracellular matrix
