# Supplementary material for: Role of Apyrase in Mobilization of Phosphate from Extracellular Nucleotides and in Regulating Phosphate Uptake in Arabidopsis
Source: Int J Mol Sci. 2025 Dec 9;26(24):11857. doi: 10.3390/ijms262411857 (PMC12732521; doi:10.3390/ijms262411857)
Supplement: Supplementary file 1 [file ijms-26-11857-s001.zip › ijms-3964788-supplementary.pdf]

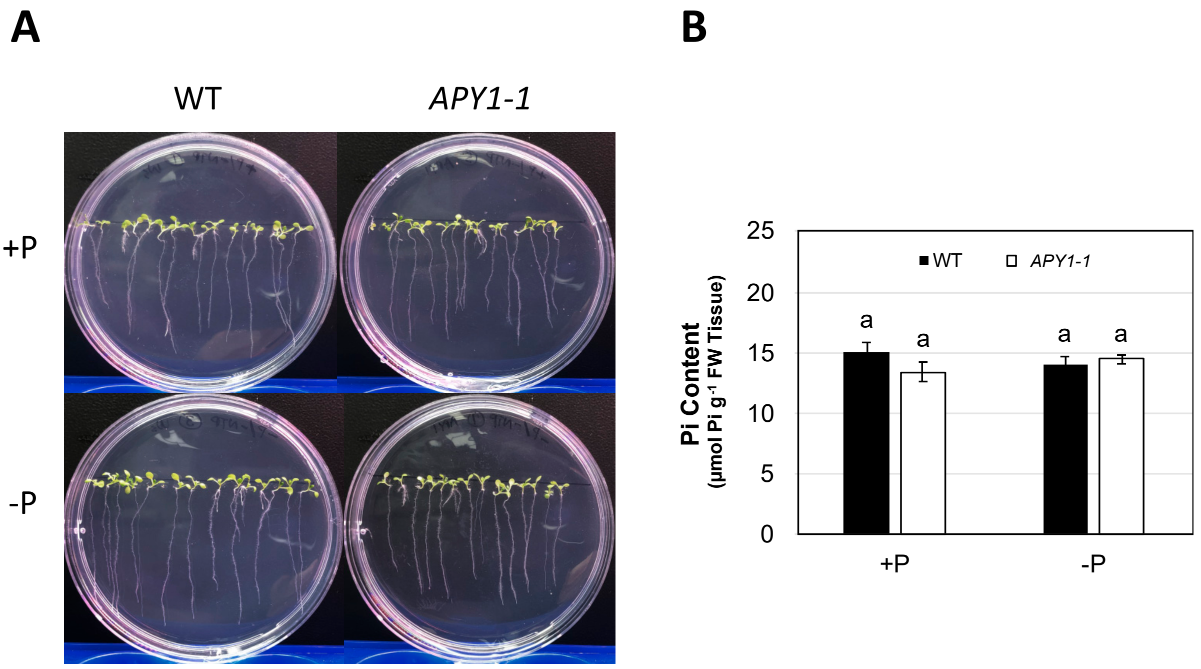

Figure S1. Plate photos of 10-day-old WT and *APY1-1* seedlings growing under P sufficiency or limitation (**A**). Seedling Pi contents (**B**) are means ±S.E for four biological replicates of 3-5 seedlings each. Lowercase letters indicate significant differences, as determined by 1-ANOVA with post-hoc Tukey honest significant difference (HSD) testing ($p \leq 0.01$).

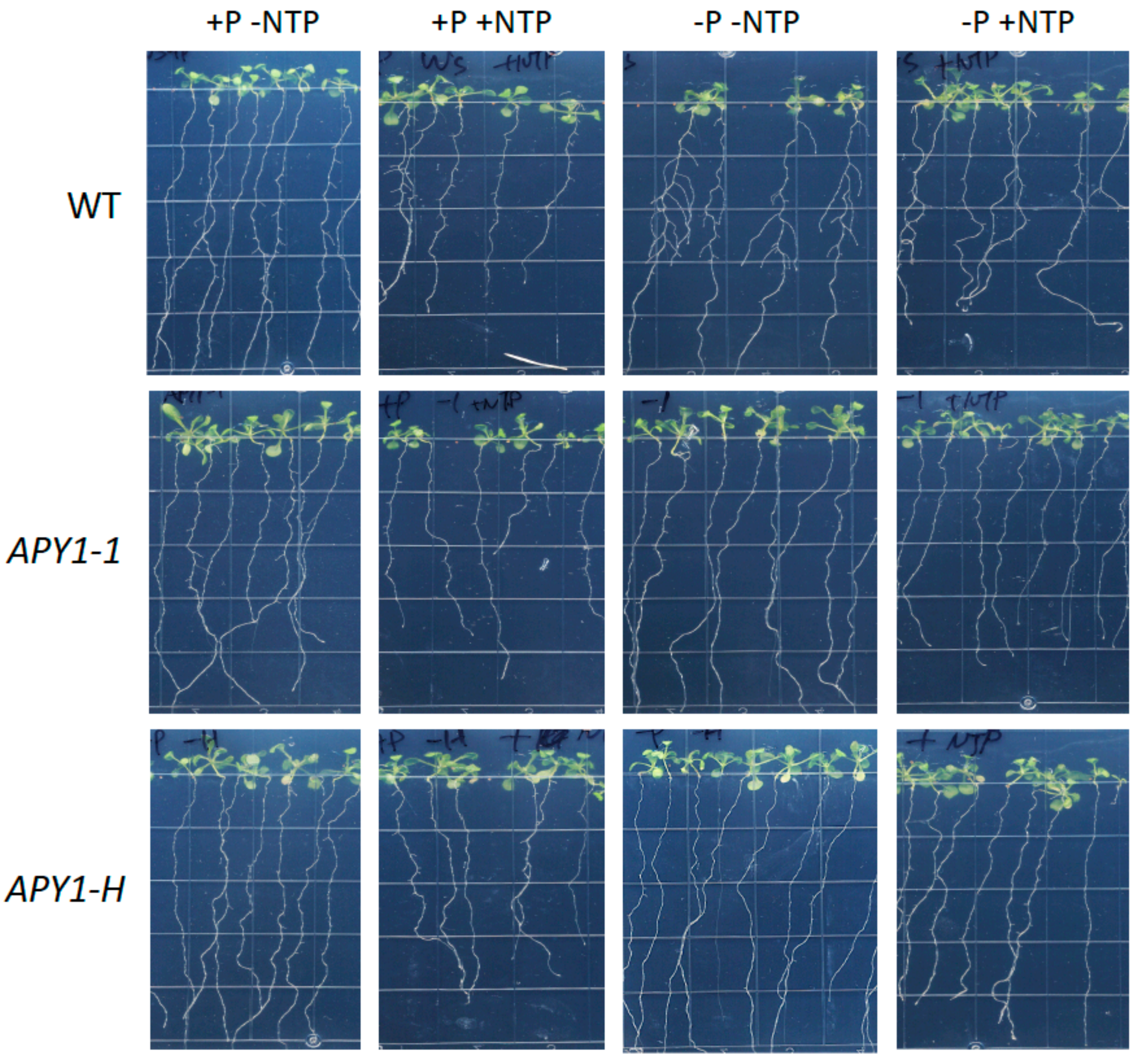

Figure S2. Effects of NTP or Pi supplementation on Arabidopsis seedlings growing on P-sufficient or P-limited medium. Seedlings were grown for 10 days on +P or -P medium, then for an additional two days, with or without either NTP supplementation. Results are typical of two independent experiments with 10-20 seedlings each. Etch lines on plates = 1 cm.

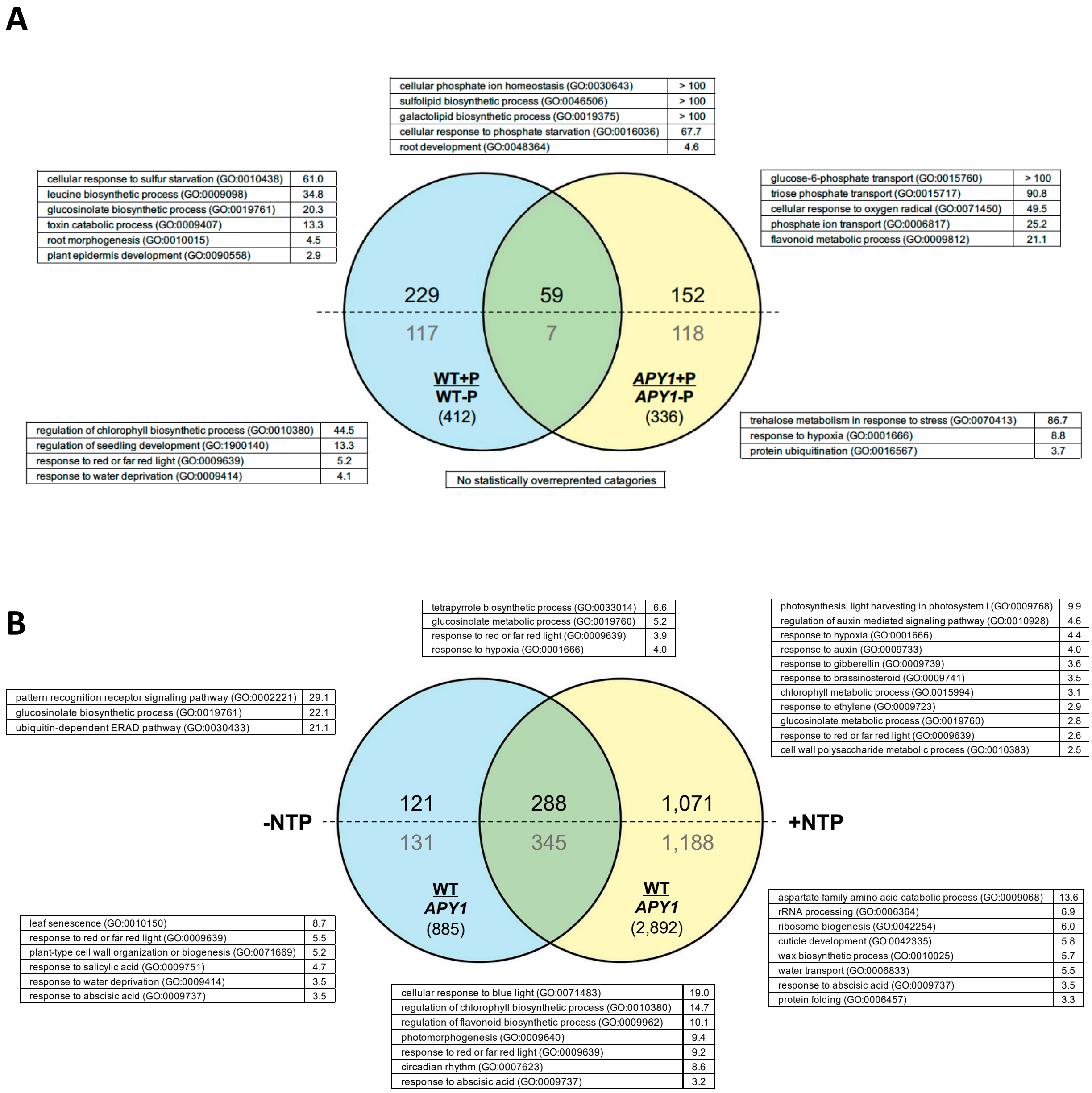

Figure S3. Gene enrichment analyses for WT and *APY1-1* seedlings in response to Pi deficiency or NTP. 15-day-old seedlings were grown on Pi-deficient or Pi-sufficient medium, without NTP supplementation (**A**) or on Pi-replete medium, with or without NTP supplementation (**B**). Numbers of DEG in each dataset are indicated in parentheses. Numbers of induced or repressed genes are indicated in black and gray, respectively. Overrepresented GO Bio Process categories and fold enrichment values for each set of DEG are shown.

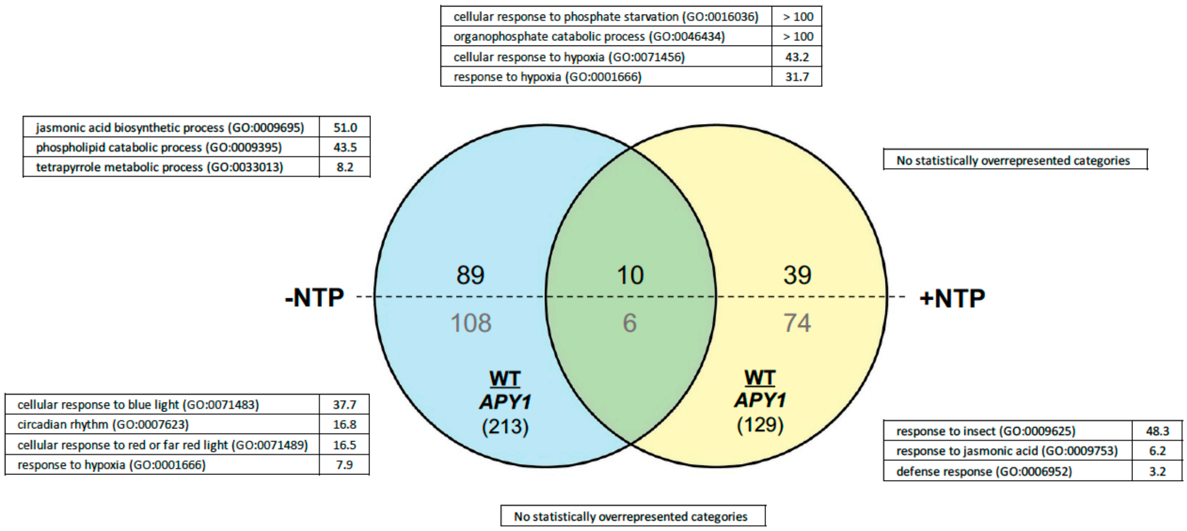

Figure S4. Gene enrichment analyses for WT and *APY1-1* seedlings growing on Pi-deficient medium, with or without NTP supplementation. Numbers of DEG in each dataset are indicated in parentheses. Numbers of induced or repressed genes are indicated in black and gray, respectively. Overrepresented GO Bio Process categories and fold-enrichment values for each set of DEG are shown.

Table S1. qRT-PCR validation of RNA-seq expression values for three phosphate starvation-responsive genes in day 15 WT and APY1 seedlings. Data are ratios of WT or *APY1-1* gene expression for P-limited seedlings, compared with seedlings growing on +P medium.

Locus ID	Gene ID	Description	RNA-seq		qRT-PCR					
			WT+P-NTP	APY1+P-NTP	WT+P-NTP			APY1+P-NTP		
			WT-P-NTP	APY1-P-NTP	WT-P-NTP			APY1-P-NTP		
					Mean	SE	p-Value	Mean	SE	p-Value
AT5G43370	PHT1;2	Inorganic phosphate transporter 1;2	2.4	5.2	5.8	2.6	0.20	36.5	5.4	0.02
AT3G02040	GDPD1	Glycerophosphodiester phosphodiesterase 1	1.5	4.3	2.7	0.5	0.06	13.2	3.5	0.07
AT5G20150	SPX1	SPX domain-containing protein 1		3.9	1.4	0.2	0.22	7.9	0.7	0.01

Table S2. Expression of genes related to phosphate starvation responses and regulation of Pi homeostasis in WT or *APY1-1* seedlings grown on media with or without Pi, ± NTP supplementation. The heatmap shows relative fold-change (FC) expression for each gene in pairwise comparisons (sample B/A ratio), which are one of two types: Same WT or *APY1-1* genotype, different experimental treatments (green), or WT versus *APY1-1* responses to the same experimental treatment (blue).

AGI	Sample	Relative Expression (FC)								Symbol	Annotation 1
	A	WT+P-NTP	APY1+P-NTP	WT+P-NTP	APY1+P-NTP	WT+P-NTP	WT+P+NTP	WT-P-NTP	WT-P+NTP		
	B	WT-P-NTP	APY1-P-NTP	WT+P+NTP	APY1+P+NTP	APY1+P-NTP	APY1+P+NTP	APY1-P-NTP	APY1-P+NTP		
**Phosphate Transport**											
AT3G47420			2.1		-1.6		-2.6			G3Pp1	Glycerol-3-phosphate permease 1
AT4G25220		2.0								G3Pp2	Glycerol-3-phosphate permease 2
AT4G17550						-1.5	-1.9			G3Pp4	Glycerol-3-phosphate permease 4
AT3G52190			1.6							PHF1	Phosphate transporter traffic facilitator1
AT3G23430							-1.5			PHO1	PHOSPHATE 1
AT1G68740			2.1							PHO1-H1	Phosphate transporter PHO1 homolog 1
AT1G35350							1.7			PHO1-H8	Phosphate transporter PHO1 homolog 8
AT2G33770				1.5	1.6					PHO2,UBC2	Phosphate 2; ubiquitin-conjugating enzyme E2 24
AT5G43350		1.5	2.2		-1.8		-1.5			PHT1;1	Inorganic phosphate transporter 1;1
AT5G43370		2.4	5.2		-2.0		-2.3			PHT1;2	Inorganic phosphate transporter 1;2
AT2G38940		1.7	1.8		-1.8			1.8		PHT1;4	Inorganic phosphate transporter 1;4
AT2G32830			1.6							PHT1;5	Inorganic phosphate transporter 1;5
AT1G76430			1.7							PHT1;9	Inorganic phosphate transporter 1;9
AT1G63010							-1.5			VPT1	Vacuolar phosphate transporter 1
**Pi Mobilization**											
AT1G14250					1.7		4.7			APY5	Apyrase 5
AT3G02040		1.5	4.3		-3.1		-1.7	1.7	2.4	GDPD1	Glycerophosphodiester phosphodiesterase 1
AT4G29690					-1.8					NPP3	Ecto-nucleotide pyrophosphatase / alkaline phosphodiesterase 3
AT1G13750		1.6	1.8							PAP1	Purple acid phosphatase 1 (inactive)
AT1G25230			-1.9			1.6	2.8			PAP4	Purple acid phosphatase 4; secreted
AT2G01890					-1.7		-1.6			PAP8	Purple acid phosphatase 8; secreted
AT2G16430							-1.5			PAP10	Purple acid phosphatase 10; cytoplasmic, secreted
AT2G27190		1.7	1.7							PAP12	Purple acid phosphatase 12; secreted
AT2G46880		3.5						1.7		PAP14	Purple acid phosphatase 14; secreted (inactive)
AT3G17790		1.9	2.5		-1.9		-2.4		2.0	PAP17	Purple acid phosphatase 17; secreted
AT3G52820			2.1							PAP22	Purple acid phosphatase 22; secreted
AT4G36350			1.5							PAP25	Purple acid phosphatase 25; secreted
AT1G73010		1.6	2.5		-2.7			1.8	2.3	PPA1	Inorganic pyrophosphatase 1
AT1G17710		2.4	2.6		-2.9			1.7	2.8	PPA2	Inorganic pyrophosphatase 2
AT2G46860			1.6							PPA3	Inorganic pyrophosphatase 3
AT3G53620			1.9					1.5		PPA4	Inorganic pyrophosphatase 4
AT4G01480		1.5	1.7							PPA5	Inorganic pyrophosphatase 5
AT2G02990				-1.8	-1.9				-1.7	RNS1	Ribonuclease 1
AT1G14210		1.6									Ribonuclease T2, cell wall (secreted)
AT4G29270							1.5				HAD superfamily, subfamily IIIB acid phosphatase
AT2G39920							2.1				HAD superfamily, subfamily IIIB acid phosphatase
AT5G44020			-1.5								HAD superfamily, subfamily IIIB acid phosphatase
**Pi Signaling, Regulation of Phosphate Starvation Responses**											
AT2G38170							2.0			CAX1	Vacuolar cation/proton exchanger 1
AT1G25550						1.7	1.9			HHO3	Transcription factor HRS1 HOMOLOG 3
AT1G13300					-2.2		-1.9			HRS1	HYPERSENSITIVITY TO LOW PI-ELICITED PRIMARY ROOT SHORTENING 1
AT3G09922		3.3	1.5		-1.6		-5.5			IPS1	INDUCED BY PI STARVATION 1
AT5G03545		2.8	2.5	-2.0	-3.5		-1.9			IPS2/AT4	INDUCED BY PI STARVATION 2
AT2G43010						3.6	3.8	2.0		PIF4	Transcription factor phytochrome interacting factor 4
AT5G04190			-1.6			2.3	3.0			PKS4	Phytochrome kinase substrate 4
AT5G20150			3.9		-2.0		-2.1	1.8	2.0	SPX1	SPX domain-containing protein 1
AT2G26660			1.8							SPX2	SPX domain-containing protein 2
AT2G45130		5.0	2.5					2.4		SPX3	SPX domain-containing protein 3
AT5G04340							-2.5			ZAT6	Zinc finger protein ZAT6
**Phosphate Starvation Responsive**											
AT4G35090		-1.6				-2.2	-2.3			CAT2	Catalase-2
AT1G20620						1.7	2.3			CAT3	Catalase-3
AT3G08040					-1.8		-3.3			DTX43	DETOXIFICATION 43
AT4G08950						1.8	2.3			EXO	Phosphate-responsive 1 family protein EXORDIUM
AT1G35140							1.8			EXL1	Phosphate-responsive 1 family protein; EXORDIUM-like 1
AT5G09470			2.4					1.7		PUMP6	Mitochondrial uncoupling protein 6
AT1G53310			1.5							PPC1	Phosphoenolpyruvate carboxylase 1
AT4G02270		2.5	1.5		-1.8					RHS13	Root hair specific 13
AT3G15990							-1.9			SULTR3;4	Sulfate transporter 3.4
**Inositol Pyrophosphate Synthesis**											
AT4G33770		1.5	1.7							IPTK2	Inositol 1,3,4-triphosphate 5/6 kinase 2
AT3G01310							1.8			VIP/VIH1	Inositol hexakisphosphate kinase
**Phospholipid Remodeling**											
Phospholipid catabolism											
AT3G05630		1.7	2.5					1.6		PLPZETA2	Phospholipase D zeta 2
AT3G18000		2.1							1.5	PEAMT1	Phosphoethanolamine N-methyltransferase 1
Sulfolipid synthesis											
AT5G01220		2.3	3.1					1.8	1.6	SQD2	SULFOQUINOVOSYLDIACYLGLYCEROL 2
AT4G33030		1.6	2.1					1.5		SQD1	SULFOQUINOVOSYLDIACYLGLYCEROL 1
Galactolipid synthesis											
AT5G20410		2.4	2.6							MGD2	Monogalactosyldiacylglycerol synthase 2
AT2G11810		2.8	2.4		-1.6			2.4		MGD3	Monogalactosyldiacylglycerol synthase 3

Table S3. Expression of flavonoid biosynthesis genes in WT or *APY1-1* seedlings grown on media with or without Pi, ± NTP supplementation. The heatmap shows relative fold-change (FC) expression for each gene in pairwise comparisons (sample B/A ratio), which are one of two types: Same WT or *APY1-1* genotype, different experimental treatments (green), or WT versus *APY1-1* responses to the same experimental treatment (blue).

AGI	Sample	Relative Expression (FC)								Symbol	Annotation 1
	A	WT+P-NTP	APY1+P-NTP	WT+P-NTP	APY1+P-NTP	WT+P-NTP	WT+P+NTP	WT-P-NTP	WT-P+NTP		
	B	WT-P-NTP	APY1-P-NTP	WT+P+NTP	APY1+P+NTP	APY1+P-NTP	APY1+P+NTP	APY1-P-NTP	APY1-P+NTP		
**Flavonones**											
AT5G13930			2.3			-1.8	-1.7			CHS	Chalcone synthase
AT3G55120							-1.9			CHI1	Chalcone-flavonone isomerase 1
AT5G66230		1.6								CHI2	Chalcone-flavonone isomerase 2
AT5G05270			2.0			-1.5	-1.5			CHI3	Chalcone-flavonone isomerase 3
**Dihydroflavonols**											
AT3G51240			1.7			-1.6	-2.6			F3H	Flavanone 3-hydroxylase
**Flavonols**											
AT5G08640		1.6	1.7							FLS1	Flavonol synthase/flavanone 3-hydroxylase 1
AT5G63600							-1.5		-1.6	FLS5	Flavonol synthase/flavanone 3-hydroxylase 5
**Anthocyanins**											
AT5G42800		Not DE								DFR	Dihydroflavonol 4-reductase
AT4G22870										ANS	Anthocyanidin synthase
AT3G29590							2.3			5MAT	Malonyl-CoA:anthocyanidin 5-O-glucoside-6''-O-malonyltransferase
AT4G34135							-2.4			UGT73B2	UDP-glucosyl transferase 73B2
AT2G36790							1.6			UGT73C6	UDP-glycosyltransferase 73C6
AT5G17050			1.5			-1.9	-2.5			UGT78D2	UDP-glycosyltransferase 78D2
AT1G06000			1.6				-1.9			UGT89C1	UDP-glycosyltransferase 89C1
**Regulation of flavonoid synthesis**											
AT2G26170							-1.7			MAX1	MORE AXILLARY BRANCHES 1
AT4G38620							-1.7			MYB4	Transcription factor MYB4
AT2G47460							-2.0			MYB12	Transcription factor MYB12
AT3G46130					-1.8		-2.4			MYB48	Transcription factor MYB48
AT5G49330			1.6							MYB111	Transcription factor MYB111
**Flavonoid transport**											
AT4G25640							-1.5			DTX35	DETOXIFICATION 35

Table S4. Expression of genes involved in auxin metabolism, transport, signaling, responses and regulation of auxin homeostasis in WT or *APY1-1* seedlings grown on media with or without P, ± NTP supplementation. GO BioProcess categories for genes are indicated. The heatmap shows relative fold-change (FC) expression for each gene in pairwise comparisons (sample B/A ratio), which are one of two types: Same WT or *APY1-1* genotype, different experimental treatments (green), or WT versus *APY1-1* responses to the same experimental treatment (blue).

AGI	Sample	Relative Expression (FC)								Symbol	Annotation 1	**auxin metabolic process** (GO:0009850, GO:009851, GO:0010249, GO:0010600)	**auxin homeostasis** (GO:0010252)	**auxin transport** (GO:0060918, GO:0080162, GO:0009926, GO:0010315, GO:0010328, GO:2000012)	**auxin signaling** (GO:0009734, GO:0010928)	**response to auxin** (GO:0009733)
	A	WT+P-NTP	APY1+P-NTP	WT+P-NTP	APY1+P-NTP	WT+P-NTP	WT+P+NTP	WT-P-NTP	WT-P+NTP							
	B	WT-P-NTP	APY1-P-NTP	WT+P+NTP	APY1+P+NTP	APY1+P-NTP	APY1+P+NTP	APY1-P-NTP	APY1-P+NTP							
AT1G28010						-1.7	-3.0			ABCB14	ABC-type transporter 14					
AT3G28345					-1.8		-2.8			ABCB15	ABC-type transporter 15			■		
AT1G59870				-1.5						ABCB36	ABC-type transporter 36			■	■	■
AT2G22810					1.7					ACS4	1-Aminocyclopropane-1-carboxylate synthase 4					■
AT5G13300							1.5			AGD3	ARF GTPase-activating protein					■
AT4G12550				1.7	-2.6		-5.5			AIR1	Lipid-binding protein AIR1					■
AT1G08980			-1.6			1.7	2.0			AMI1	Amidase 1	■				
AT2G46530							1.6			ARF11	Auxin response factor 11					■
AT3G61830							1.6			ARF18	Auxin response factor 18					■
AT1G19220					1.6		2.0			ARF19	Auxin response factor 19					■
AT3G59900		1.8								ARGOS	Auxin-Regulated Gene involved in Organ Size					■
AT2G38120							1.5			AUX1	AUXIN RESISTANT 1			■		■
AT5G12050					2.7	1.7	3.6			BG1	BIG GRAIN 1-like D			■		
AT1G54200					1.7		2.0			BG2	BIG GRAIN 1-like B			■		
AT3G13980					1.5	1.6	2.2			BG3	BIG GRAIN 1-like A			■		
AT5G63160					1.8	-2.8	-1.7	-2.5		BT1	BTB/POZ domain-containing protein 1					■
AT3G48360			-2.7	-1.7			1.5	-2.4		BT2	BTB/POZ and TAZ domain-containing protein 2					■
AT5G67480		1.5					1.6			BT4	BTB/POZ domain-containing protein 4					■
AT4G37610			-1.7							BT5	BTB and TAZ domain protein 5					■
AT3G55120							-1.9			CHI1	Chalcone--flavonone isomerase 1					■
AT5G13930			2.3			-1.8	-1.7			CHS	Chalcone synthase; CHS			■		■
AT3G25717				1.6	1.8	1.8	2.1			DVL6	DEVIL 6					■
AT2G30420							1.8			ETC2	ENHANCER OF TRY AND CPC 2					■
AT3G45970			-1.8				1.9			EXLA1	Expansin-like A1					■
AT3G55500							-3.9			EXPA16	Expansin A16					■
AT5G08640		1.6	1.7							FLS1	Flavonol synthase/flavanone 3-hydroxylase					■
AT5G63600							-1.5		-1.6	FLS5	Flavonol synthase/flavanone 3-hydroxylase 5					■
AT5G56860		-1.7	-1.5							GATA21	GATA transcription factor 21					■
AT4G26150		-1.7				-2.2	-2.0	-1.6		GATA22	GATA transcription factor 22					■
AT4G37390					1.9		2.6			GH3.2	Indole-3-acetic acid-amido synthetase GH3.2		■			■
AT2G23170					1.9					GH3.3	Indole-3-acetic acid-amido synthetase GH3.3		■			
AT4G27260					1.8		2.1			GH3.5	Indole-3-acetic acid-amido synthetase GH3.5		■			■
AT5G54510					3.0		3.8			GH3.6	Indole-3-acetic acid-amido synthetase GH3.6		■		■	■
AT5G13370						-1.8	-3.0			GH3.12	Indole-3-acetic acid-amido synthetase GH3.12	■				
AT5G47370					1.8		2.2			HAT2	Homeobox-leucine zipper protein HAT2				■	■
AT4G16780					1.6		2.3			HAT4	Homeobox-leucine zipper protein HAT4					■
AT5G67060							2.2			HEC1	Transcription factor HECATE 1			■		
AT4G14560					2.1		2.4			IAA1	AUX/IAA transcriptional regulator IAA1					■
AT1G04240							2.1			IAA3	AUX/IAA transcriptional regulator IAA3					■
AT1G52830					2.3		2.4			IAA6	AUX/IAA transcriptional regulator IAA6					■
AT3G23050					1.6		1.6			IAA7	AUX/IAA transcriptional regulator IAA7					■
AT1G04550						-1.5				IAA12	AUX/IAA transcriptional regulator IAA12/BDL					■
AT4G14550					2.3		1.9			IAA14	AUX/IAA transcriptional regulator IAA14					■
AT3G15540					1.8					IAA19	AUX/IAA transcriptional regulator IAA19					■
AT5G25890							-1.8			IAA28	AUX/IAA transcriptional regulator IAA28					■
AT4G32280					2.3		4.8			IAA29	AUX/IAA transcriptional regulator IAA29					■
AT1G44350							1.6			ILL6	IAA-amino acid hydrolase ILR1-like 6	■				
AT4G27280							2.3			KRP1	Calcium-binding EF-hand family protein					■
AT5G47390						1.6	1.5			KUA1	Transcription factor KUODA1		■			■
AT1G08410							-1.7			LSG1-2	GTPase LSG1-2			■		
AT2G26170							-1.7			MAX1	MORE AXILLARY BRANCHES 1			■		
AT3G10870					1.5		1.6			MES17	Methylesterase 17	■				
AT5G05500		1.7			-2.0					MOP10	Pollen Ole e 1 allergen and extensin family protein					■
AT2G47460							-2.0			MYB12	Transcription factor MYB12					■
AT3G28910						1.6	2.3			MYB30	Transcription factor MYB30					■
AT4G37260							2.5			MYB73	Transcription factor MYB73				■	
AT3G50060					1.5		2.8			MYB77	Transcription factor MYB77				■	
AT1G56010					-1.6					NAC001	NAC domain containing protein 1				■	
AT5G39610							-2.0			NAC006	NAC domain containing protein 6					■
AT5G56750						1.6	1.8		1.6	NDL1	N-MYC downregulated-like 1			■		■
AT5G11790			1.6							NDL2	N-MYC downregulated-like 2			■		
AT4G31820							1.8			NPY1	NAKED PINS IN YUC MUTANTS 1			■		
AT4G07410							-1.7			PCN	Transducin family protein / WD-40 repeat family protein			■		
AT2G43010						3.6	3.8	2.0		PIF4	Transcription factor phytochrome interacting factor 4	■			■	
AT3G59060							1.6			PIF5	Transcription factor phytochrome interacting factor 5	■			■	
AT2G17500					-1.8		-1.8		-1.5	PILS5	Auxin efflux carrier family protein; PIN-LIKES 5		■	■		■
AT2G01420					1.5		2.3			PIN4	Auxin efflux carrier component 4		■	■		
AT1G23080					1.9		2.7			PIN7	Auxin efflux carrier component 7		■	■		
AT3G05630		1.7	2.5					1.6		PLPZETA2	Phospholipase D zeta 2					■
AT4G39403							2.7			PLS	POLARIS		■	■		■
AT5G48900		1.5			2.0		1.9			PLY20	Pectate lyase 20					■
AT3G07010					1.5		2.1			PLY8	Pectate lyase 8					■
AT3G30350					1.7		2.2			RGF4	Root meristem growth factor 4			■		
AT4G16515					2.8		2.9			RGF6	Root meristem growth factor 6			■		■
AT5G64770					1.5					RGF9	Root meristem growth factor 9			■		■
AT5G17300			-1.9		-1.9		-2.8	-1.8		RVE1	REVEILLE 1	■			■	
AT4G34770			-1.7			2.1	4.6			SAUR1	Auxin-responsive protein SAUR1					■
AT4G34790						1.9	4.1			SAUR3	Auxin-responsive protein SAUR3					■
AT4G34810		1.7								SAUR5	Auxin-responsive protein SAUR5					■
AT2G21200					2.2		2.2			SAUR7	Auxin-responsive protein SAUR7					■
AT2G18010							2.5			SAUR10	Auxin-responsive protein SAUR10					■
AT4G38825					1.8		5.0			SAUR13	Auxin-responsive protein SAUR13					■
AT4G38850					1.8		1.7			SAUR15	Auxin-responsive protein SAUR15					■
AT4G38860					1.7		2.0			SAUR16	Auxin-responsive protein SAUR16					■
AT5G18010					1.9		3.0			SAUR19	Auxin-responsive protein SAUR19			■		
AT5G18020						-1.8				SAUR20	Auxin-responsive protein SAUR20					■
AT5G18030					1.9		2.5			SAUR21	Auxin-responsive protein SAUR21					■
AT5G18050					3.0		4.6			SAUR22	Auxin-responsive protein SAUR22					■
AT5G18060					2.4		3.3			SAUR23	Auxin-responsive protein SAUR23					■
AT5G18080					2.5		2.5			SAUR24	Auxin-responsive protein SAUR24					■
AT3G03850					2.4		2.2			SAUR26	Auxin-responsive protein SAUR26					■
AT3G03840					1.9		1.8			SAUR27	Auxin-responsive protein SAUR27					■
AT3G03830							3.4			SAUR28	Auxin-responsive protein SAUR28			■		■
AT3G03820					1.8		2.4			SAUR29	Auxin-responsive protein SAUR29					■
AT5G53590							-2.1			SAUR30	Auxin-responsive protein SAUR30					■
AT4G31320		1.6								SAUR37	Auxin-responsive protein SAUR37					■
AT2G37030							2.4			SAUR46	Auxin-responsive protein SAUR46					■
AT4G34750							2.1			SAUR49	Auxin-responsive protein SAUR49					■
AT4G34760					1.5		1.7			SAUR50	Auxin-responsive protein SAUR50					■
AT3G53250					2.2		4.3			SAUR57	Auxin-responsive protein SAUR57					■
AT3G60690							-1.6			SAUR59	Auxin-responsive protein SAUR59					■
AT1G20470		-1.6				-2.8	-3.0	-1.5		SAUR60	Auxin-responsive protein SAUR60					■
AT1G29440					1.9		1.7			SAUR63	Auxin-responsive protein SAUR63			■		
AT1G29450					2.0		2.7			SAUR64	Auxin-responsive protein SAUR64					■
AT1G29460					1.8	1.7	2.7			SAUR65	Auxin-responsive protein SAUR65					■
AT1G29500				1.5	1.8		1.7			SAUR66	Auxin-responsive protein SAUR66					■
AT1G29510					1.7		1.7			SAUR67	Auxin-responsive protein SAUR67					■
AT5G10990							-2.1			SAUR69	Auxin-responsive protein SAUR69					■
AT5G20820							2.8			SAUR76	Auxin-responsive protein SAUR76					■
AT1G70560					1.5		1.8			TAA1	Tryptophan aminotransferase of Arabidopsis 1	■			■	
AT1G05680						-2.0	-3.5			UGT74E2	UDP-glycosyltransferase 74E2	■				
AT4G15550			1.5			-1.8	-3.4			UGT75D1	UDP-glycosyltransferase 75D1	■				
AT3G29370					2.2	1.7	3.8			UPBEAT	Transcription factor UPBEAT-like protein					■
AT1G75500							1.6			WAT1	Walls Are Thin 1, WAT1			■		
AT5G57560							3.7			XTH22	Xyloglucan endotransglucosylase/hydrolase 22					■
AT4G28720					1.9		2.6			YUC8	Flavin-binding monooxygenase family protein	■				
AT1G07135							3.1				Glycine-rich protein					■
AT4G23820					1.6		1.9				Pectin lyase-like superfamily protein					■
AT4G14819					2.2		2.8				Protein of unknown function (DUF1677)					■

Table S5. Expression of genes involved in root hair initiation and elongation in WT or *APY1-1* seedlings grown on media with or without P, ± NTP supplementation. The heatmap shows relative fold-change (FC) expression for each gene in pairwise comparisons (sample B/A ratio), which are one of two types: Same WT or *APY1-1* genotype, different experimental treatments (green), or WT versus *APY1-1* responses to the same experimental treatment (blue).

AGI	Relative Expression (FC)								Root hair initiation	Root hair elongation	Gene Symbol	Description
	Ws+P-NTP	APY1+P-NTP	Ws+P-NTP	APY1+P-NTP	Ws+P-NTP	Ws+P+NTP	Ws-P-NTP	Ws-P+NTP				
	Ws-P-NTP	APY1-P-NTP	Ws+P+NTP	APY1+P+NTP	APY1+P-NTP	APY1+P+NTP	APY1-P-NTP	APY1-P+NTP				
AT1G19220				1.6		2.0				+	ARF19	Auxin response factor 19
AT3G25250					-1.7				+	+	OXI1	OXIDATIVE SIGNAL-INDUCIBLE 1
AT2G38120						1.5				+	AUX1	AUXIN RESISTANT 1
AT4G39400						1.6				+	BRI1	BRASSINOSTEROID INSENSITIVE 1
AT2G27050				1.5		2.2			+	+	EIL1	ETHYLENE INSENSITIVE 3-like 1
AT5G61350						-2.7				+	CAP1	$[Ca^{2+}]_{cyt}$-associated protein kinase
AT5G51810						2.1				−	GA20ox2	Gibberellin 20 oxidase 2
AT3G28910					1.6	2.3				+	MYB30	Transcription factor MYB30

Table S6. Expression of genes related to nucleotide metabolism and apoplastic salvaging of NTP in WT or *APY1-1* seedlings grown on media with or without P, ± NTP supplementation. The heatmap shows relative fold-change (FC) expression (sample B/A ratio) for each DEG in pairwise comparisons for *APY1-1* versus WT seedlings.

AGI	Symbol	Annotation	Sample	Relative FC Expression (Mean Ratio Sample A/B)			
			A	WT+P-NTP	WT+P+NTP	WT-P-NTP	WT-P+NTP
			B	APY1+P-NTP	APY1+P+NTP	APY1-P-NTP	APY1-P+NTP
***De Novo* Synthesis**							
AT3G54470	UMPS	UMP Synthase (bifunctional)			-1.5		
AT4G18440	ASL	Adenylsuccinate lyase		1.6	1.9		
AT3G55010	AIRS	AIR synthase			-1.7		
AT4G34740	ASE2	Glutamine amidophosphoribosyltransferase 2, ATase 2			-2.0		
AT1G79470	IMPDH1	IMP dehydrogenase 1			-1.5		
AT3G21110	SAICARS	SAICAR synthetase			-1.6		
**Synthesis of NTP and dNTP**							
AT1G30820	CTPS1	CTP synthase 1					
AT3G12670	CTPS2	CTP synthase 2			-1.6		
AT3G46940	DUT	Deoxyuridine 5'-triphosphate nucleotidohydrolase					
AT4G25280	UMK2	UMP/CMP Kinase 2		-1.5			
AT5G35170	AMK5	AMP kinase 5			1.5		
AT3G01820	AMK7	AMP kinase 7		-1.6	-2.0		
AT2G41880	GMK1	GMP kinase 1		-1.5			
AT4G09320	NDPK1	Nucleoside diphosphate kinase 1					
**Salvaging (Intracellular)**							
AT5G23070	TK1b	Thymidine kinase 1b			-2.0		
AT1G72040	dNK	Deoxynucleoside kinase					
**Catabolism**							
AT3G08860	PYD4	β-alanine-pyruvate aminotransferase 3			6.0		
AT4G04955	ALN	Allantoinase			2.9		
AT4G34900	XDH2	Xanthine dehydrogenase 2 (inactive)		1.6	1.7		
**Salvaging (Extracellular)**							
Nucleases							
AT2G02990	RNS1	Ribonuclease 1					-1.7
Nucleotidases							
AT1G14250	APY5	Apyrase 5			4.7		
Transporters (PM-localized) for Cellular Uptake of Nucleobases, Nucleosides							
AT1G19770	PUP14	Purine permease 14		1.6	2.1		
AT1G57990	PUP18	Purine permease 18			3.1		

Table S7. Primers used for verification of WT and *APY1* lines by PCR genotyping and qRT-PCR quantification of *APY1* expression or validation of DEG in RNA-seq datasets.

Primer Name	Sequence (5'->3')
Ws Genotyping	
AT5G42320-Col0-F	CAAATATGGTTACCATTTCTTCGTTCACAAAGAACT
AT5G42320-Ws2-F	AAATATGGTTACCATTTCTTCGTTCACAAAGATGA
AT5G42320-R	TTTTGACAGATGATTCCTTTTCTTCCAGACTTTT
qRT-PCR	
PP2A-F	GTTGTGTGAGCACGCAAAGA
PP2A-R	ACCCTCGATCAACATAATCACCC
APY1-1-F	CGTCCGTCTCTGTCATCGAG
APY1-1-R	CAGTTGCCCCAACTCTGACA
PHT1;2-F	TGCAGGATACCCACCAGGTA
PHT1;2-R	GCACTCACGGCAGTAAGACT
GDPD1-F	ATGTCTCGGAAGTGCCAAGC
GDPD1-R	CGTACACCACTCCCTGTTCC
SPX1-F	GTGTGTTTTCATTGCCGCCT
SPX1-R	TGGAATAGAGCGAGTGTGCC
